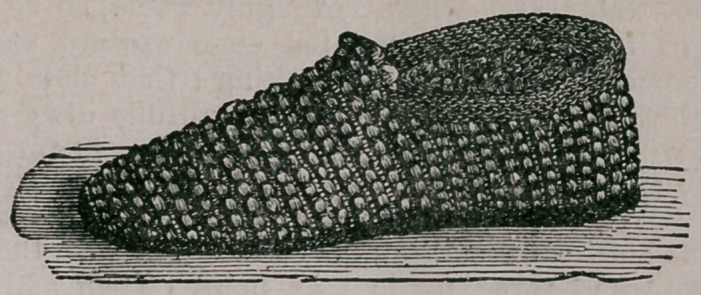# Household

**Published:** 1887-08

**Authors:** 


### HOUSEHOLD.

There is no article of dress that adds more to the comfort of the wearer, than a light, loosely fitting slipper, especially in the long winter evenings ; for invalids, it is not only a luxury, but a positive necessity. To be able to make such a slipper is an accomplishment that always goes with thanks. The cut represents such a piece of work, and to make it the following directions will be found complete :

*Knitting Material Required*: Four ounce blue and four ounce white Berlin wool ; four pins No. 12 (Walker's gauge), and a pair of cork soles.

Commence the slipper at the toe with blue wool, cast on ten stitches, increase by putting the wool over the pin at beginning of each row to make a stitch. When knitting with the white wool take it from two balls so as to have two lengths.

1st Row : Knit plain.

2nd Row : Make one, knit one*, take the double white wool, turn it twice over the pin to form a loop of about three-quarters of an inch (see design), with the left hand pin pass the last knitted loop over the four loops of white, knit two, repeat from * to the end of the row.

3d Row : Make one at the beginning of the row, slip the loops of white wool, knit the blue ; in knitting the blue stitch, pass the blue wool with which you are knitting, round the double white wool ; in knitting the next stitch, this will draw up the white wool close to the work, and so carry it to the other side to be ready for working the next row of loops.

4th Row : Make one, knit the blue stitches plain, knit the four white loops at the back as one stitch.

5th Row : Make one, knit to the end of the row. Repeat from second row, increasing at the beginning of each row until the work is wide enough across the instep.

Now divide the stitches for the sides, casting off ten in the center ; with the third pin continue to work on the side stitches, as before, without increase or decrease, until you have the length from the instep to the back of the heel, then cast off and work the other side in the same way ; sew the two sides together at the back with a needle and wool.

Now pick up the stitches round the top of slipper, on three pins, and with a fourth pin and blue wool, knit ten rows, cast off, turn this plain piece over, and hem it down to the top of inside of slipper to form a roll round the edge. Sew the bottom of slipper neatly and firmly to a strong cork sole lined with wool.

Bottling Eggs.—The office of the air-tight fruit jar may be enlarged to preserve eggs as well as fruit. As soon as the eggs are collected, put the jars into hot water, and when thoroughly warm, so as to rarefy the air, put the eggs in the jar, the pointed ends upward, and pack them with paper or something, to prevent breaking ; then close the jar before taking it out of the water. If the work is skillfully done and the jar is tight, the eggs will keep for many months and be as fit for the breakfast table as the day they were laid.

Bishop's Bread.—Beat fourteen ounces of sugar with the yolks of six eggs and the whites of three, for half an hour. Then add slowly eight ounces of flour, six ounces of blanched almonds cut in thin strips, six ounces of raisins and three ounces of citron cut in fine pieces. Pour in a well-greased pan and bake slowly.

Lamb Chops a la Tartare—Salt and flour the chops, fry in nice dripping and set over hot water to keep warm ; heat in a saucepan a cupful of good broth, well-skimmed, thicken with browned flour, season with pepper and salt and stir in a heaping tablespoonful of capers, or, if you cannot get them, the same quantity of chopped pickles. Boil up once, pour on the chops and let them stand over boiling water ten minutes before they go to the table.

Tea Cake.—Mix for fifteen minutes, four eggs with half a pound of sugar, half a grated nutmeg and as much powdered cloves as will lie on the tip of a dinner knife. Then add half a pound of dry and sifted flour, and mix thoroughly ; have a greased or waxed tin, drop a tablespoonful of the dough at intervals upon it, and bake a pale brown in a moderate oven.

CORN BREAD.

Two cups of Indian, one cup wheat,
One cup sour milk, one cup sweet,
One good egg, that well you beat.
Half a cup molasses, too,
Half cup sugar add thereto,
With one spoon of butter new ;
Salt and soda, each a spoon,
Mix up quickly and bake it soon,
Then you'll have corn bread complete,
Best of all corn bread you meet.

Boiled Indian Pudding.—This is improved for some people if suet is added to give it richness. Chop a quarter of a pound of beef suet very fine, add an equal quantity of sugar, one teaspoonful of ginger, half a teaspoonful of salt, enough sweet milk to moisten the meal, and a teaspoonful of baking powder, or about a cup of sour milk and a teaspoonful of soda. This should boil in a bag for at least three hours, and be served hot, with wine sauce.

## Figures and Tables

**Figure f1:**